# Long-Term Immunomodulatory Impact of VNS on Peripheral Cytokine Profiles and Its Relationship with Clinical Response in Difficult-to-Treat Depression (DTD)

**DOI:** 10.3390/ijms25084196

**Published:** 2024-04-10

**Authors:** Erhan Kavakbasi, Evelien Van Assche, Kathrin Schwarte, Christa Hohoff, Bernhard T. Baune

**Affiliations:** 1 Department of Psychiatry, University Hospital Münster, University of Münster, Albert-Schweitzer-Campus 1, Building A9, 48149 Münster, Germanyhohoffc@ukmuenster.de (C.H.); bernhard.baune@ukmuenster.de (B.T.B.); 2 Department of Psychiatry, Melbourne Medical School, The University of Melbourne, Melbourne, VIC 3052, Australia; 3 The Florey Institute of Neuroscience and Mental Health, The University of Melbourne, Parkville, VIC 3010, Australia

**Keywords:** difficult-to-treat depression, treatment-resistant depression, vagus nerve stimulation, cytokines, inflammation, interleukins

## Abstract

Vagus nerve stimulation (VNS) represents a long-term adjunctive treatment option in patients with difficult-to-treat depression (DTD). Anti-inflammatory effects have been discussed as a key mechanism of action of VNS. However, long-term investigations in real-world patients are sparse. In this naturalistic observational study, we collected data on cytokines in peripheral blood in *n* = 6 patients (mean age 47.8) with DTD and VNS treatment at baseline and at 6 months follow-up. We have identified clusters of peripheral cytokines with a similar dynamic over the course of these 6 months using hierarchical clustering. We have investigated cytokine changes from baseline to 6 months as well as the relationship between the cytokine profile at 6 months and long-term response at 12 months. After 6 months of VNS, we observed significant correlations between cytokines (*p* < 0.05) within the identified three cytokine-pairs which were not present at baseline: IL(interleukin)-6 and IL-8; IL-1β and TNF-α; IFN-α2 and IL-33. At 6 months, the levels of all the cytokines of interest had decreased (increased in non-responders) and were lower (5–534 fold) in responders to VNS than in non-responders: however, these results were not statistically significant. VNS-associated immunomodulation might play a role in long-term clinical response to VNS.

## 1. Introduction

Depression is one of the most common reasons for disability in the world [1]. Approximately 16–18% of people experience at least one episode during their lifespan, often resulting in a decline in the quality of life and functioning [2]. About one third of patients with depression do not adequately respond to antidepressant pharmacotherapy [3]. These patients may need neuromodulatory approaches, such as electroconvulsive therapy (ECT), repetitive transcranial magnetic stimulation (rTMS), or vagus nerve stimulation (VNS) in addition to pharmacotherapy to respond to treatment or achieve remission. In particular, for patients with a high burden of disease following a chronic course of difficult to treat depression (DTD), VNS has been shown to improve the quality of life and disease management, although complete and sustained remission remains difficult to achieve [4,5]. VNS requires the implantation of a pulse generator in the left breast region, which is connected to stimulation electrodes on the left vagal nerve through a metal lead [6]. VNS has received approval for the long-term management of depression both in the EU and the USA [6]. The Canadian guidelines recommend VNS as a long-term option due to its maintenance efficacy [7]. VNS has a delayed onset of antidepressant action, with its first clinical benefits often being seen beyond 6–12 months [6].

Peripheral inflammation has been described as a potential pathophysiological mechanism in the development of major depressive disorder and other psychiatric disorders [8]. Elevated proinflammatory cytokine levels have been observed in the blood of individuals with depression [8]. Hence, treatment strategies aiming to control peripheral inflammation have received attention in the treatment of depression in recent years, e.g., the addition of celecoxib, an anti-inflammatory agent to an oral antidepressant [1,9,10]. The vagus nerve is thought to modulate the interaction between peripheral immune activation, e.g., in the blood, and the central nervous system [11]. Anti-inflammatory effects are considered a core feature of the antidepressant mechanism of action of VNS [12]. The vagus nerve is part of the neuro–immune axis. In the case of an inflammatory process in peripheral tissue, pro-inflammatory cytokines can bind to interleukin receptors on the vagal nerve and activate vagal afferents [13]. This information leads to activation of the nucleus of the solitary tract, which in turn activates neurons to release corticotrophin-releasing factor. Activation of the hypothalamus–pituitary–adrenal axis and the release of cortisol lead to an anti-inflammatory response [13]. Additionally, vagal efferents inhibit the release of peripheral pro-inflammatory cytokines [13]. The vagal neurotransmitter acetylcholine has been shown to inhibit the release of pro-inflammatory cytokines, mainly TNF-α in macrophages, which has been described as the efferent “cholinergic anti-inflammatory pathway” [14]. This reaction also takes place in the macrophages of the spleen and is mediated by nicotinic acetylcholine receptors [13,15]. Due to its anti-inflammatory properties, VNS has also been investigated in a variety of inflammatory diseases, such as inflammatory bowel disease [16]. Anti-inflammatory properties were also described for antidepressant treatments other than VNS, seen to influence the levels of cytokines, such as TNF-α, in peripheral blood [17].

Previous research has focused on describing an increase or decrease in various cytokines in individuals with depression. The most common cytokines which have been associated with depression are interleukin-1 (IL-1), IL-6, and tumor necrosis factor alpha (TNF-α) [18]. Meta-analytic data have revealed that cytokines such as IL-7, IL-8, IL-12. and IL-18 were higher in depression patients than in controls [19]. In contrast, other studies have yielded inconsistent and contradictory results on whether a certain cytokine is increased or decreased in depressed individuals [1]. It is most likely that altered cytokine levels during the course of depression serve as an unspecific indicator of immune activation in depressed individuals [11]. Furthermore, it is known that cytokines act as a network with complex interaction among themselves [20], making it challenging to create an explanatory model through the description of single molecules alone. For instance, IL-1 has been found to induce the transcription of IL-6, which in turn stimulates C-reactive protein (CRP) production [21,22]. Immune activation also involves changes in immune cell types and differentiation, leading to the up- and downregulation of various cytokines at the same time [20].

Current understandings of the anti-inflammatory effects of VNS are typically derived from animal models, in vitro experiments, or transcutaneous VNS [14,23,24], yielding inconclusive results. However, data from long-term studies involving real world patients, both for invasive and transcutaneous VNS, are sparse. Recently, a long-term pilot observation in *n* = 6 VNS patients revealed decreased levels of IL-7, CCL2 (CC-chemokine ligand), and other CCL proteins after 4 years of VNS, appealing to the immunomodulatory effects of the treatment [25]. However, efforts to explain the interplay between peripheral inflammation and depression by describing changes in certain singular cytokines have failed to provide an adequate model. The relationship between peripheral inflammation and depression is likely more complex, requiring new approaches beyond those describing changes in particular cytokines.

The objective of the present work is to integrate the information of 14 jointly acting cytokines through hierarchical clustering. Due to the delayed antidepressant action of VNS, we hypothesized that there are changes between cytokine profiles at baseline and in the early cytokine profiles of patients after 6 months of treatment. We expect to identify clusters of cytokines that act synergistically and to describe the correlation of the cytokines in the clusters at baseline and after 6 months as a primary analysis. As part of an exploratory analysis, we have investigated changes in the cytokines in these clusters from baseline to 6 months in responders and non-responders to VNS. Furthermore, we anticipate that these cytokine profiles at 6 months are related to the corresponding clinical response status at 12 months, as clinical experience and the literature repeatedly showed that only after about 12 months of VNS depressive symptoms are improved to such an extent that the distinction between responders and non-responders becomes increasingly reliable [26]. Therefore, we investigated the levels of cytokines at 6 months in responders compared to non-responders to VNS at 12 months as part of a second exploratory analysis.

## 2. Results

### 2.1. Sample Description

**Figure 1:**
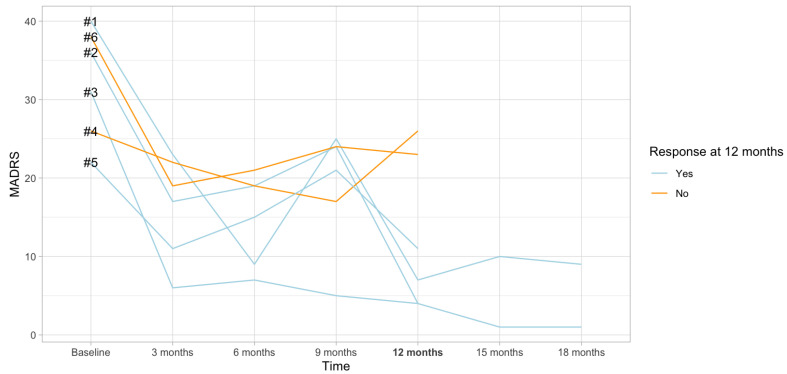
Figure shows the MADRS evolution over the course of the follow-up period. The numbers indicate individual cases.

A total of *n* = 6 patients have been included in this study. Their clinical characteristics are described in Table 1. All the patients suffered from severe difficult-to-treat depression (DTD). The mean duration of their current episode was more than two years (28.7 months), indicating the severe course of the disease. Patients were usually first diagnosed with depression in young adulthood (mean age at first onset 21.2). The baseline MADRS score was 32.2 and decreased to 12.5 after 12 months of VNS treatment. The response rate at 12 months was 66.6%, and 4 out of 6 patients fulfilled the response criteria at the 12-month follow-up visit (at least 50% reduction in MADRS score). Figure 1 provides the changes in MADRS score over time in responders and non-responders.

### 2.2. Cytokine Clusters

On the cytokine level, the dendrogram for cytokines at 6 months suggests that these are the three separate clusters of cytokines with the closest pairs (Figure S4B):

- 0.336 between IL-1β and TNF-α, indicating the closest relationship;
- 0.344 between IL-6 and IL-8;
- 0.599 between IFN-α2 and IL-33.

**Figure 2:**
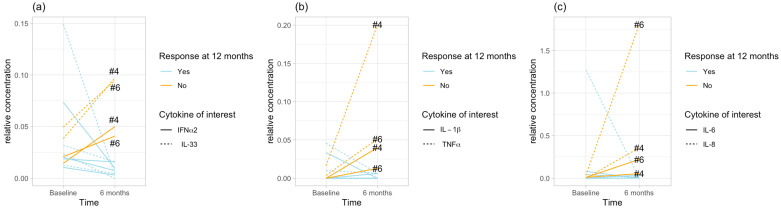
Changes in cytokine concentrations from baseline to 6 months visit. The cytokine concentration tended to decrease in responders and increase in non-responders to VNS. (**a**–**c**) Demonstrate the respective cytokine clusters. The numbers (4, 6) indicate the two non-responders to VNS. The blue lines indicate the individuals, who responded to VNS at 12 months.

Interestingly, this clustering of cytokines was not present at baseline, and it occurred following 6 months of VNS treatment. Non-parametric testing to validate these correlations confirmed this result after 6 months of treatment, as well as confirming the absence of this correlation at baseline. There was a significant correlation at 6 months, whereas at baseline the pairs were not correlated to each other. The correlation analysis between the cytokines for each cluster and for both timepoints is provided in Table 2. Figure 2a–c shows the changes in the cytokine pairs from baseline to 6 months.

### 2.3. Cytokines and Clinical Response

**Figure 3:**
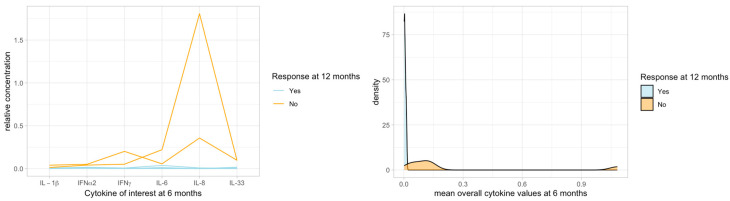
All cytokines of interest had much lower concentrations at 6 months in responders to VNS than in non-responders at 12 months. These differences at 6 months between responders and non-responders were not statistically significant, probably due to the small sample size (*p* = 0.133 for each cytokine). On the left figure, each line indicates one individual case.

The cytokine levels tended to increase from baseline to 6 months in non-responders responders to VNS at 12 months and to decrease in responders (Table 3). However, these changes were not statistically significant. Overall, the levels of all six cytokines combined of interest showed a bi-modal distribution at 6 months, corresponding to the response status to VNS at 12 months: cytokines at 6 months were lower in responders at 12 months than in non-responders (Table 4, Figure 3). These differences were not statistically significant.

## 3. Discussion

In this study, we investigated the changes in cytokine profiles in peripheral blood during VNS and their relationship with clinical response to VNS over the long-term course of depression in individuals with DTD and a high burden of disease. Our approach highlights the importance of incorporating the network capacities of cytokines and acknowledging their internal relationships into future models. We observed three cytokine pairs that were not correlated at baseline but which, after a rearrangement during the VNS treatment, showed a highly significant correlation. The cytokine pairs of interest with high correlation at 6 months identified in this study were as follows: Il-6 and IL-8; IL-1β and TNF-α; IFN-α2 and IL-33. This observation indicates that chronic stimulation of the vagus nerve might modulate the profiles of peripheral cytokines and might harmonize and regulate a previously disturbed immune reaction. These cytokines were probably modulated during VNS treatment and might be involved in the anti-inflammatory mechanism of action of VNS. Dysregulated cytokine profiles in patients with depression have been described previously [27,28]. However, previous studies have focused on the elevation or depletion of singular cytokines, and thus a variety of cytokines have been reported to be altered [27,28]. To the best of our knowledge, our approach is the first to describe the alteration within clusters of cytokines rather than in singular cytokines.

Anti-inflammatory effects have been suggested as a main mechanism of action of VNS, [13] previously characterized by two anti-inflammatory pathways. Firstly, activation of the hypothalamus–pituitary–adrenal axis leads to the secretion of cortisol [13], which exerts its anti-inflammatory effects broadly [29]. Irregularities in cortisol secretion have been found to be predictive of elevated CRP levels (C reactive protein) [30]. In addition, circadian changes in cortisol levels have been observed to modulate the relationship between reported stress and dysregulated inflammatory biomarkers, such as IL-6 [31]. Secondly, activation of the cholinergic anti-inflammatory pathway inhibits the secretion of TNF-α in the macrophages of the spleen [13]. Further, cytokines such as IL-1β, IL-6, and IL-18 are involved in efferent vagal anti-inflammatory pathways [32]. The immunomodulatory effects of VNS were reported recently in *n* = 6 patients [25]. The authors described a decrease in the levels of IL-7 as well as various CCL proteins in VNS patients after 4 years of VNS, but they did not differentiate between cytokine profiles in responders and non-responders. Furthermore, in contrast to our study, they did not observe relevant changes in IL-1β, IL-6, and TNF-α [25]. Nevertheless, both the aforementioned report and our study indicate that VNS may be associated with peripheral immunomodulation. However, it may not be appropriate to describe the anti-inflammatory effects of VNS treatment by investigating single cytokines. Instead, functional interactions were described between cytokines, e.g., between IL-1β and TNF-α; IL-1β and IL-6; IL-6 and CRP; or TNF-α and monocyte chemotactic protein 1 (MCP-1, also known as CCL2) [21,22,33,34]. Our study suggests that IL-1β and TNF-α as well as IL-6 and IL-8 are closely correlated to each other following VNS treatment, whereas there was an uncorrelated and probably disturbed mechanism of action between these cytokines at baseline before VNS treatment. Overall, these results suggest that VNS modulates cytokine profiles in peripheral blood early in the course of treatment, and this effect might even be related to long-term clinical response to VNS at 12 months. Furthermore, the cytokine profiles at 6 months seem to have the potential to differentiate future responders from non-responders at 12 months. In our study, cytokine levels decreased from baseline to 6 months in the later responders to VNS, while there was an increase in these levels in the later non-responders. According to the results of our study, the investigation of peripheral cytokine profiles early in the course of VNS may help to distinguish later responders from non-responders to treatment. This might indicate a VNS-associated modification of the imbalanced, dysregulated baseline inflammatory cytokine profile towards a more normalized profile with balanced interplay after 6 months of VNS therapy. Re-balanced or rearranged correlation might lead to further downstream modifications in the periphery and the brain. In particular, brain modifications such as reorganization and changes in connectivity or neuroplasticity might need longer timespans (e.g., [35]) and might involve TNF signaling [36]. In line with this, our observed IL-1β and TNF-α correlation after 6 months of VNS treatment is the most strongly clustered. In the present study, the concentrations of all the cytokines of interest was consistently higher at 6 months in future (12 months) non-responders than in the patients responding to VNS, reflecting anti-inflammatory VNS action in responders. However, these differences in cytokine levels at 6 months between responders and non-responders, as well the changes in cytokine levels from baseline to 6 months, were not statistically significant, probably due to the small sample size, and should therefore be treated with caution. Our results are in line with the clinical observation of delayed antidepressant action of VNS. In the VNS registry, the median time to first response in the VNS group was 12 months [26]. Our results suggest that anti-inflammatory and immunomodulatory changes in peripheral blood following VNS may occur far earlier than clinical response to VNS.

Immunomodulatory effects and changes in cytokines profiles have also been reported following other neuromodulatory interventions in depression. In the case of the rapid-acting antidepressant ketamine, a randomized trial showed a rapid decrease in TNF-α levels following ketamine infusion, which also correlated to the alleviation in depression severity [37]. Electroconvulsive therapy has been shown to have acute pro-inflammatory effects after a single session, whereas later in the course a decrease in inflammatory activity has been observed [38,39]. Transcranial magnetic stimulation was also found to reduce levels of peripheral cytokines in depressed individuals [40]. Immunomodulatory effects seem to occur across different treatment techniques. However, VNS is a long-term treatment, which, contrary to other techniques, has a late onset of antidepressant efficacy [6]. Detecting immunomodulatory effects early in the course of treatment and prior to clinical response may be particularly useful in patients treated with VNS. Studies comparing the immunomodulatory effects of VNS with the effects of other techniques are lacking.

Interestingly, the number of women enrolled in this study was disproportionately higher than the number of men. One possible explanation for this observation is that depression occurs twice as frequently in women as in men [41]. Furthermore, the prevalence of recurrent and chronic courses of depression are more frequent in women than in men [41]. The small sample size may have led to an exaggeration of the sex differences in our study. One has to bear in mind that the disproportionate sex differences may affect the transferability of the results to male depression cases.

Our results are preliminary and should be interpreted with caution due to the small sample size and uncontrolled design, but they provide an encouraging approach for severely affected patients. In contrast to the majority of previous VNS studies involving animal models or in vitro investigations [42], our study is one of the first to investigate treatment responses in real-world patients according to cytokine profiles and clusters rather than single cytokines. Given the complex interplay between cytokines in the complex pathophysiological mechanism of depression [20], this approach to investigate clusters of cytokines may be more useful and appropriate than attempting to predict responses to anti-depressant treatment based on single cytokines. There is probably a closer relationship and a synergistic mode of action between cytokines which have been clustered together. It may be useful to determine the clusters of cytokines which interact in the mechanism of depression and which change during treatment course.

## 4. Strengths and Limitations

This study has several strengths and limitations. The small sample size is the main limitation of this study. Another important limitation in this study is that disproportionately more women were enrolled than men, which may constrain the transferability of the results to male depression. Furthermore, this is an uncontrolled and naturalistic study, thus we are unable to state whether the immunomodulatory effect and rearrangement in peripheral cytokines observed in this investigation were a VNS-specific effect or an unspecific phenomenon associated with depression treatment and clinical improvement in general. Apart from VNS, the concomitant treatments administered to the patients may have had an impact on the pathophysiological mechanism of their disease and changes in indicators of hypothesized peripheral inflammation in depression. Our results need to be confirmed in a larger controlled study, with the aim of predicting later response to VNS through early investigations of peripheral cytokines. Given the long-term nature of VNS treatment and its delayed antidepressant action, prediction of treatment response is particularly valuable in this group of patients with a severe and often chronic course of disease. Given the lack of other robust biological predictors of VNS treatment response, our results are encouraging. Further studies may focus on the prediction of treatment response based on early investigations of peripheral cytokine profiles and may pave new ways in VNS research.

## 5. Material and Methods

### 5.1. Study Design

The patients (*n* = 6) were recruited at the Department of Psychiatry, University Hospital of Münster, Germany. Patients with chronic (>2) or recurrent (minimum 2 prior episodes) major depression were included in this naturalistic observational VNS study, whose current episode had not responded to an adequate number of treatment attempts [43]. After baseline assessment pre-surgery, follow-up examinations with a clinical assessment and blood sample collection were conducted every 3–6 months. Depression symptom severity was assessed by using the Montgomery–Åsberg Depression Rating Scale (MADRS). The decision to conduct VNS treatment was made in a naturalistic design independent of this study. Ethics approval was obtained by the institutional review board and the local ethics commission in Münster, Germany, for the clinical study as well as a local amendment for the collection and analysis of blood (2019-475-b-S, (ClinicalTrials.gov NCT03320304)).

### 5.2. Inflammatory Protein Measurements

Peripheral blood was taken into 9 ml CAT serum vacuettes to derive serum after incubation (30 min. at room temperature), centrifugation (10 min. at 2500× *g*), aliquoting (300 µL portions), and storing (immediately at −80 °C) for later protein analyses.

The inflammatory protein hsCRP was measured in the central laboratories of the university hospital Muenster, Germany, by utilizing immunonephelometry, using serum together with Siemens reagent N Cardio Phase TM hsCRP (cat. no. OQIY13, product no. 10446090) on a Siemens BN II system (both by Siemens Healthcare GmbH, Erlangen, Germany). Further, 13 inflammatory proteins (IL-1β, IFN-α2, IFN-γ, TNF-α, MCP-1, IL-6, IL-8, IL-10, IL-12p (70), IL-17A, IL-18, IL-23, and IL-33) were measured in serum (in triplicate, 1:2 dilution, distributed over two analysis plates) by using an LEGENDplex™ bead-based immunoassay (Human Inflammation Panel1; BioLegend, San Diego, CA, USA; Cat. No. 740809) following the manufacturer’s instructions. The serum samples of patients were randomized on plates based on the patients’ sex and age, whereas all samples of the same patient (at different timepoints) were run on the same plate (for within-individual analyses). For quality control, additional serum samples (in-house positive controls) spiked with known concentrations of standards (BioLegend) were included on each plate. For quantitative cytometric fluorescence analysis, all samples were acquired on a flow cytometer (CytoFLEX S; Beckman Coulter, Krefeld, Germany) in accordance with the manufacturer’s instructions. BioLegend software Qognit version 2022-07-15 (BioLegend, San Diego, CA, USA) was used to calculate final cytokine concentrations, which were normalized to the in-house positive controls, resulting in relative concentrations.

### 5.3. Statistical Analyses

All cytokines were scaled in preparation for the analyses. Due to the small sample size, individual cytokine values were not transformed for the planned analysis or additionally manipulated in any way. Density plots are shown in the Supplementary Data (Figures S1–S3).

First, we performed an exploratory hierarchical clustering analysis using the hclust (‘stats’ package) function in R ([44]; version 4.3.1) based on Euclidian distances with particular interest in cytokines after 6 months of treatment. Clustering was performed using the Ward’s minimum variance method (Ward.D2) to identify combinations of cytokines showing the most similar patterns at a particular timepoint. Clusters at 6 months of treatment were identified by visual inspection and supported by the dendrogram height (Figure S4B). For the validation analysis, we selected the three closest pairs (distance < 0.6; total dendrogram height = 4.9; Figure S4B). A similar cluster analysis for baseline data was performed as a reference (Figure S4A). Following this selection, we validated the correlation of cytokines in the identified clusters as well as their change during VNS treatment using non-parametric Spearman’s rho correlation as a primary analysis. As part of an exploratory investigation, we examined the changes in the cytokines from baseline to 6 months in responders and non-responders to VNS at 12 months. Furthermore, we tested whether the cytokine concentrations at 6 months were higher in later non-responders to VNS than in responders as a second exploratory analysis. For non-parametric statistical tests, we used SPSS Statistics (Version 28.0.1.1., IBM, Armonk, NY, USA).

## Figures and Tables

**Table 1:** All patients were implanted with VNS due to severe difficult-to-treat MDD.

Mean age at time of implantation (years)	47.8
Gender, Women	83.3%, *n* = 5
Psychiatric comorbidities	*n* = 2, Post-traumatic stress disorder
Mean age at first onset of depression (years)	21.2
Mean duration of current episode (months)	28.7
Treatment history prior to VNS Previous ECT ECT responder Previous transcranial magnetic stimulation Previous esketamine	100% (*n* = 6) 66.6% (*n* = 4) 33.3% (*n* = 2) 33.3% (*n* = 2)
Baseline MADRS (mean)	32.2
12 months MADRS (mean)	12.5
12 months response rate (%)	66.6 (4 out of 6)

**Table 2:** Top three of cytokine pairs identified by using exploratory hierarchical clustering of all 14 cytokines.

Cluster-Based * Cytokine Pairs	Cluster Tree Height **	V0 Correlation *** Coefficient; *p*-Value	V6 Correlation *** Coefficient; *p*-Value (*p*(Corrected)-Value)
TNFa–IL-1β	extra low	0.655; *p* = 0.158	0.941; *p* = 0.005, (*p*(corr) = 0.015)
IL-6–IL-8	extra low	0.213; *p* = 0.686	0.941; *p* = 0.005, (*p*(corr) = 0.015)
IFNα2–IL-33	low	0.714; *p* = 0.111	0.829; *p* = 0.042

*: for pairs identified see Supplemental Figure S4; **: lower tree height indicates closer relationship between cytokines (Supplemental Figure S4); ***: statistics: non-parametric Spearman’s rho correlation with Bonferroni correction for multiple testing (3 pairs).

**Table 3:** Exploratory investigation of the changes in cytokines from baseline to 6-month follow-up in responders and non-responders to VNS at 12 months.

	Cytokine	Concentration Baseline (Mean)	Concentation at 6 Months (Mean)	Change	Test Statistics (Wilcoxon Test)	*p*-Value
Responder to VNS at 12 months	IL-1β	0.00843	0.00165	Decrease	−0.447	0.655
	TNF-α	0.01398	0.00343	Decrease	−1.069	0.285
	IL-6	0.03175	0.01308	Decrease	−0.365	0.715
	IL-8	0.32100	0.00203	Decrease	−1.069	0.285
	IFN-α2	0.03068	0.00893	Decrease	−1.826	0.068
	IL-33	0.05418	0.00595	Decrease	−1.826	0.068
Non-responder to VNS at 12 months	IL-1β	0.00010	0.02650	Increase	1.342	0.180
	TNF-α	0.01005	0.12595	Increase	1.342	0.180
	IL-6	0.01125	0.13780	Increase	1.342	0.180
	IL-8	0.01805	1.08195	Increase	1.342	0.180
	IFN-α2	0.01760	0.04530	Increase	1.342	0.180
	IL-33	0.04395	0.09585	Increase	1.342	0.180

**Table 4:** Exploratory analysis of the differences in cytokine concentrations at 6 months between patients who responded to VNS at 12 months and non-responders.

Cytokine	Mean Concentration of the Cytokine at 6 Months		Mann–Whitney U Test	Ratio of Cytokine Concetration in Non-responders/Responders
	Responder	Non-responder	*p*-Value (Exact; Asymptotic)	
IL-1β	0.001650	0.026500	0.049; 0.133	16.1
TNF-α	0.003425	0.125950	0.064; 0.133	36.8
IL-6	0.013075	0.137800	0.064; 0.133	10.5
IL-8	0.002025	1.081950	0.049; 0.133	534.3
IFN-α2	0.008925	0.045300	0.064; 0.133	5.1
IL-33	0.005950	0.095850	0.064; 0.133	16.1

## Data Availability

The data presented in this study are available on request from the corresponding author (local privacy and legal reasons).

## Supplementary Materials

The following supporting information can be downloaded at: https://www.mdpi.com/article/10.3390/ijms25084196/s1.
