# Supplementary material for: Long-Term Immunomodulatory Impact of VNS on Peripheral Cytokine Profiles and Its Relationship with Clinical Response in Difficult-to-Treat Depression (DTD)
Source: Int J Mol Sci. 2024 Apr 10;25(8):4196. doi: 10.3390/ijms25084196 (PMC11050644; doi:10.3390/ijms25084196)
Supplement: Supplementary file 1 [file ijms-25-04196-s001.zip › ijms-2930206-supplementary.pdf]

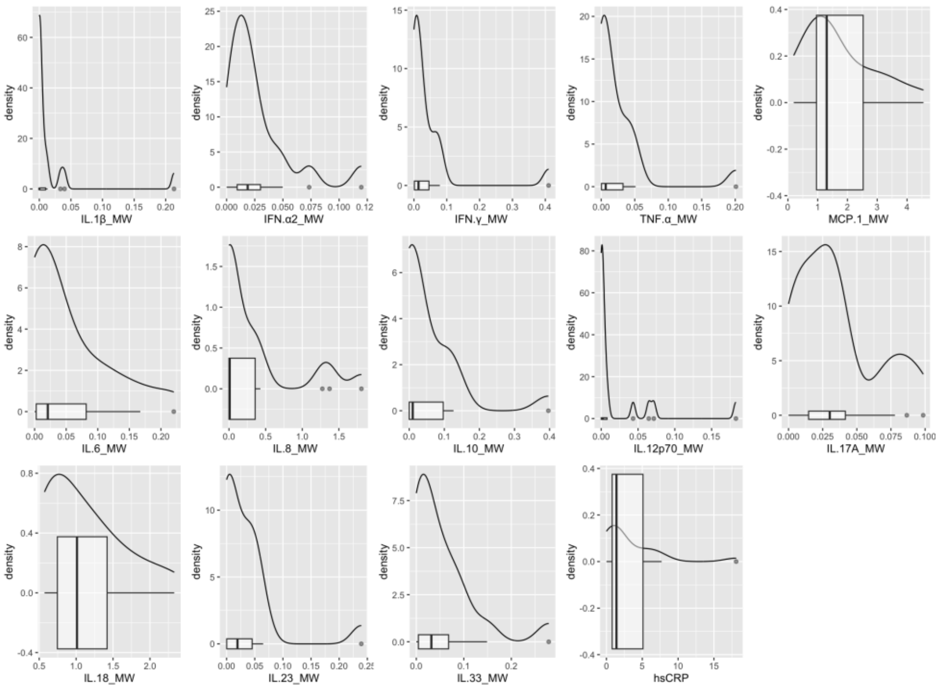

Figure S1. Density plots for methodological (wet-lab) QC: both timepoints combined. Outliers were tolerated due to the nature of the distribution of the data, the limited sample size, and the exploratory set-up.

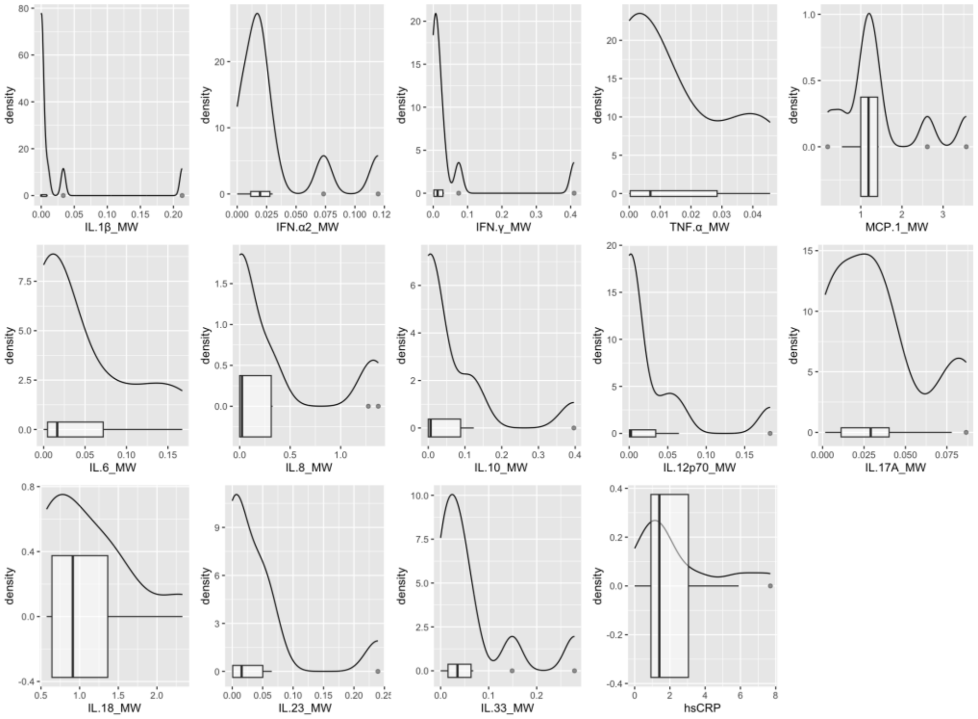

Figure S2. Density plots: cytokines at baseline.

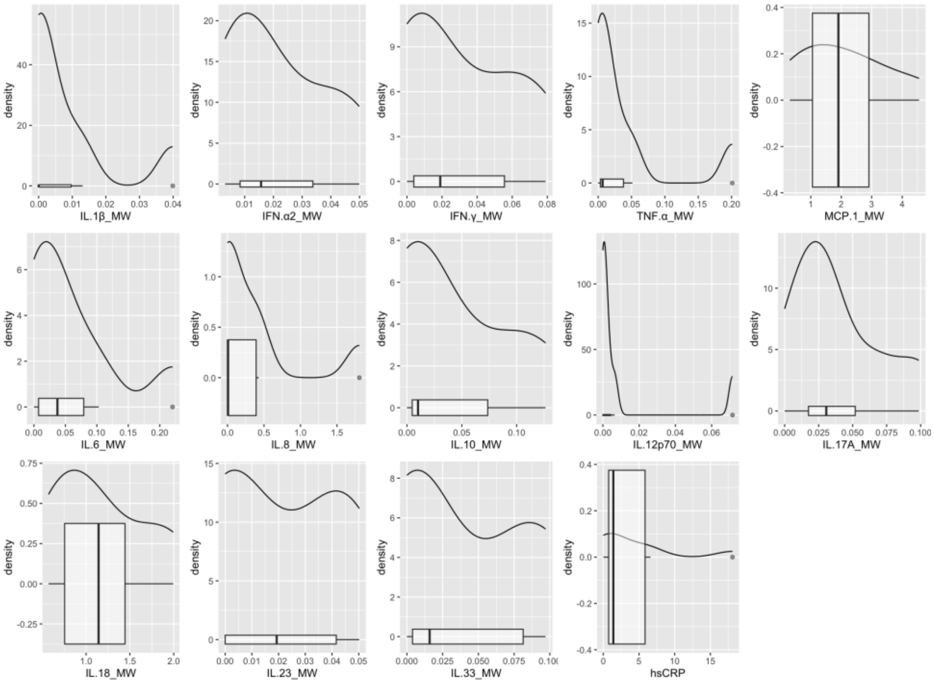

Figure S3. Density plots: cytokines at 6 months.

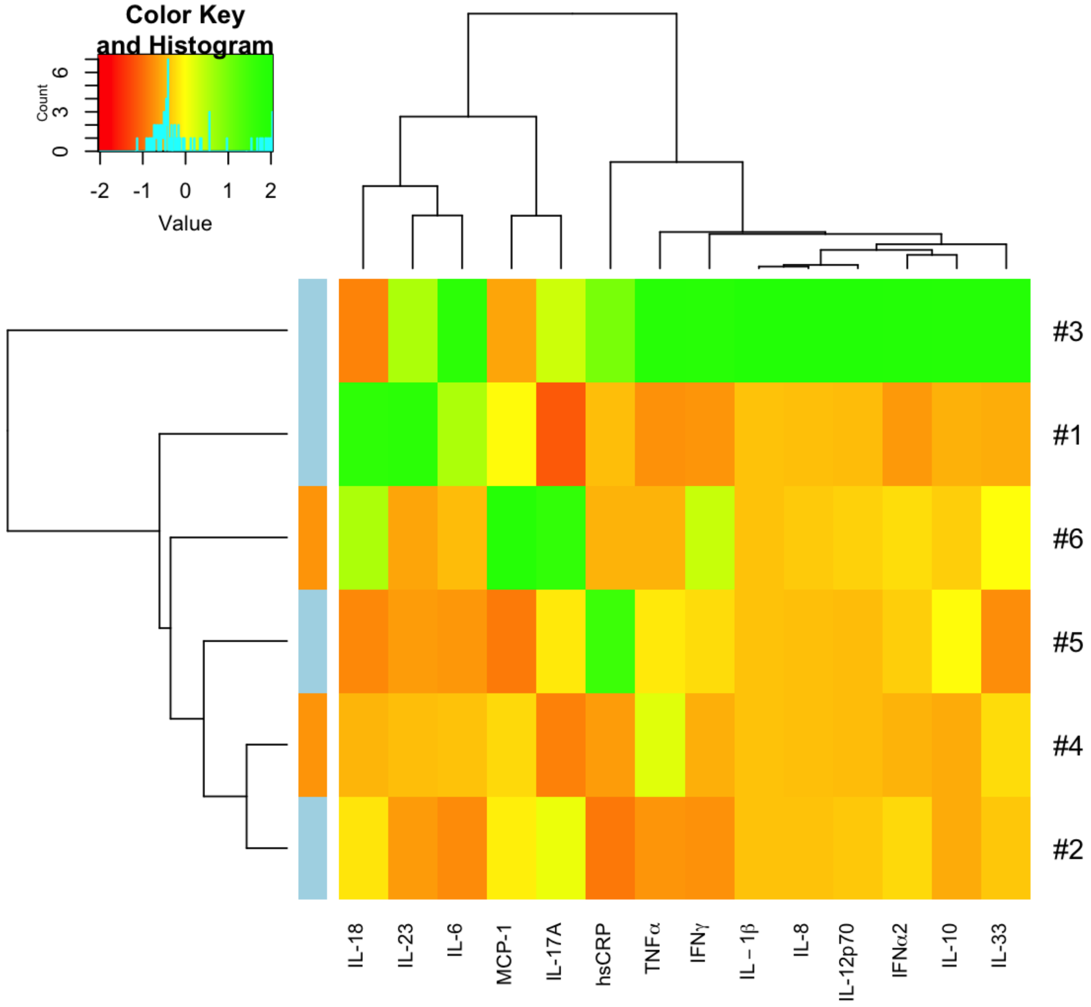

(a)

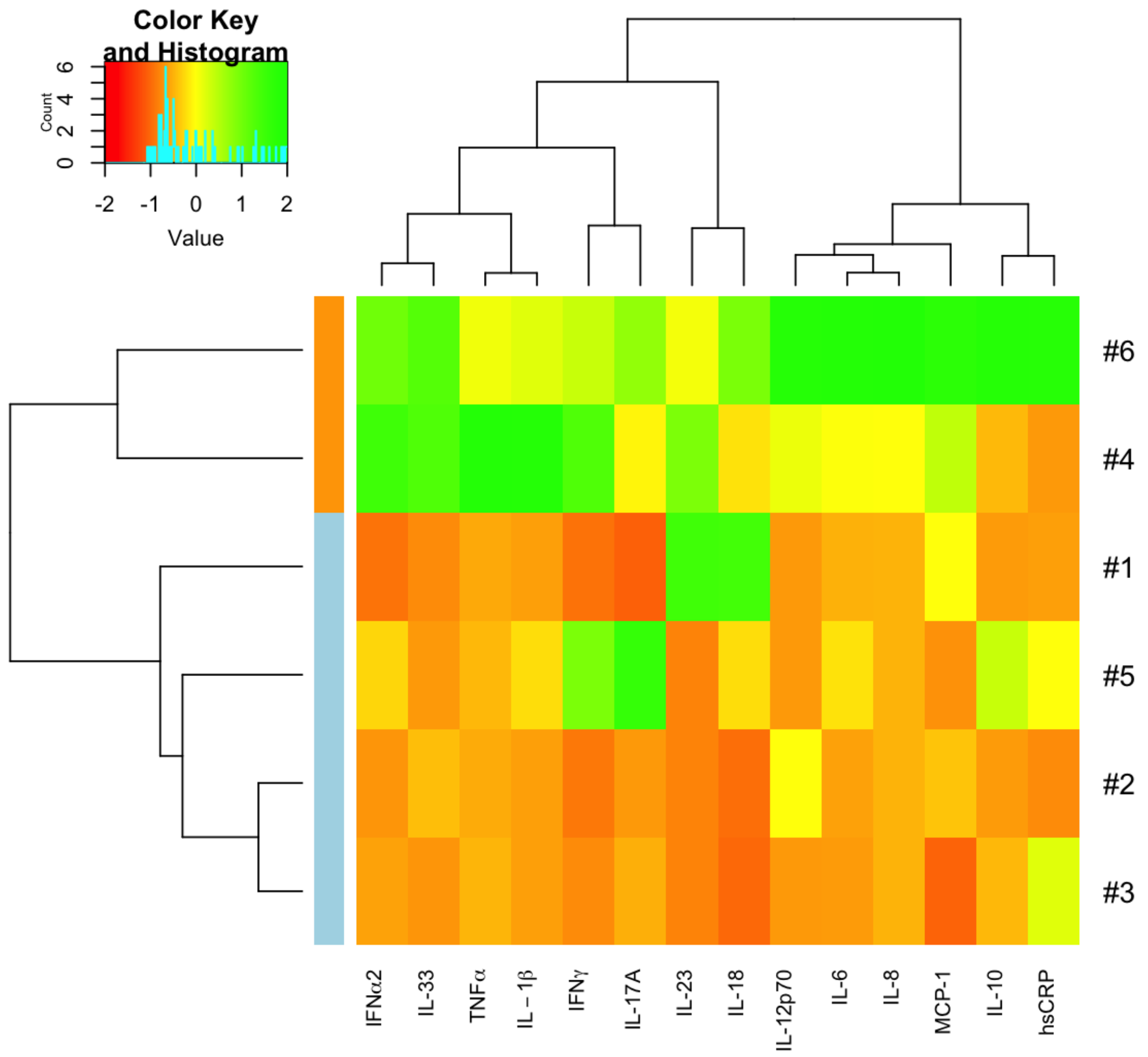

(b)

Figure S4. Exploratory clustering through hierarchical clustering methods. Exploratory heatmaps at baseline (A) and after 6 months of treatment (B). The colored side-bars in the heatmap (left) reflect the response status after 12 months of treatment (blue = response, orange = no response).
